# Treatment of allergic rhinitis during and outside the pollen season using mobile technology. A MASK study

**DOI:** 10.1186/s13601-020-00342-x

**Published:** 2020-12-09

**Authors:** A. Bédard, X. Basagaña, J. M. Anto, J. Garcia-Aymerich, P. Devillier, S. Arnavielhe, A. Bedbrook, G. L. Onorato, W. Czarlewski, R. Murray, R. Almeida, J. A. Fonseca, J. Correia da Sousa, E. Costa, M. Morais-Almeida, A. Todo-Bom, L. Cecchi, G. De Feo, M. Illario, E. Menditto, R. Monti, C. Stellato, M. T. Ventura, I. Annesi-Maesano, I. Bosse, J. F. Fontaine, N. Pham-Thi, M. Thibaudon, P. Schmid-Grendelmeier, F. Spertini, N. H. Chavannes, W. J. Fokkens, S. Reitsma, R. Dubakiene, R. Emuzyte, V. Kvedariene, A. Valiulis, P. Kuna, B. Samolinski, L. Klimek, R. Mösges, O. Pfaar, S. Shamai, R. E. Roller-Wirnsberger, P. V. Tomazic, D. Ryan, A. Sheikh, T. Haahtela, S. Toppila-Salmi, E. Valovirta, V. Cardona, J. Mullol, A. Valero, M. Makris, N. G. Papadopoulos, E. P. Prokopakis, F. Psarros, C. Bachert, P. W. Hellings, B. Pugin, C. Bindslev-Jensen, E. Eller, I. Kull, E. Melén, M. Wickman, G. De Vries, M. van Eerd, I. Agache, I. J. Ansotegui, S. Bosnic-Anticevich, A. A. Cruz, T. Casale, J. C. Ivancevich, D. E. Larenas-Linnemann, M. Sofiev, D. Wallace, S. Waserman, A. Yorgancioglu, D. Laune, J. Bousquet, the MASK study group

**Affiliations:** 1 ISGlobal, Barcelona, Spain; 2 CIBER Epidemiología y Salud Pública (CIBERESP), Barcelona, Spain; 3 Universitat Pompeu Fabra (UPF), Barcelona, Spain; 4 IMIM (Hospital del Mar Research Institute), Barcelona, Spain; 5 UPRES EA220, Pôle des Maladies des Voies Respiratoires, Hôpital Foch, Université Paris-Saclay, Suresnes, France; 6 KYomed INNOV, Montpellier, France; 7 CHU de Montpellier, Montpellier, France; 8 Medical Consulting Czarlewski, Levallois, France; 9 Research fellow, OPC, and Director, Cambridge, UK; 10 Faculdade de Medicina da Universidade do Porto, Lda Porto, Portugal; 11 Life and Health Sciences Research Institute (ICVS), School of Medicine, University of Minho, Braga, Portugal; 12 ICVS/3B’s, PT Government Associate Laboratory, Braga/Guimarães, Portugal; 13 Faculty of Pharmacy and Competence Center on Active and Healthy Ageing of University of Porto (Porto4Ageing), Porto, Portugal; 14 Allergy Center, CUF Descobertas Hospital, Lisbon, Portugal; 15 Imunoalergologia, Centro Hospitalar Universitário de Coimbra and Faculty of Medicine, University of Coimbra, Coimbra, Portugal; 16 SOS Allergology and Clinical Immunology, USL Toscana Centro, Prato, Italy; 17 Department of Medicine, Surgery and Dentistry “Scuola Medica Salernitana, University of Salerno, Salerno, Italy; 18 Division for Health Innovation, Campania Region, Federico II University Hospital Naples (R&D and DISMET), Naples, Italy; 19 CIRFF, Federico II University, Naples, Italy; 20 Department of Medical Sciences, Allergy and Clinical Immunology Unit, University of Torino, Mauriziano Hospital, Turin, Italy; 21 Unit of Geriatric Immunoallergology, University of Bari Medical School, Bari, Italy; 22 Medical School Saint Antoine, Epidemiology of Allergic and Respiratory Diseases, Department Institute Pierre Louis of Epidemiology and Public Health, INSERM, Sorbonne Université, Paris, France; 23 Allergist La Rochelle, La Rochelle, France; 24 Allergist, Reims, France; 25 Ecole Polytechnique Palaiseau, IRBA (Institut de Recherche bio-Médicale des Armées), Bretigny, France; 26 RNSA (Réseau National de Surveillance Aérobiologique), Brussieu, France; 27 Allergy Unit, Department of Dermatology, University Hospital of Zurich, Zurich, Switzerland; 28 Service Immunologie et Allergie, Centre Hospitalier Universitaire Vaudois, Lausanne, Switzerland; 29 Department of Public Health and Primary Care, Leiden University Medical Center, Leiden, The Netherlands; 30 Department of Otorhinolaryngology, Amsterdam University Medical Centres, AMC, Amsterdam, The Netherlands; 31 Clinic of Chest Diseases, Immunology and Allergology, Medical Faculty, Vilnius University, Vilnius, Lithuania; 32 Clinic of Children’s Diseases, Faculty of Medicine, Vilnius University, Vilnius, Lithuania; 33 Department of Pathology, Faculty of Medicine and Clinic of Chest Diseases, Immunology and Allergology, Faculty of Medicine, Institute of Biomedical Sciences, Vilnius University, Institute of Clinical Medicine, Vilnius, Lithuania; 34 Vilnius University Institute of Clinical Medicine, Clinic of Children’s Diseases, Department of Public Health, and Institute of Health Sciences, Vilnius, Lithuania; 35 European Academy of Paediatrics, EAP/UEMS-SP), Brussels, Belgium; 36 Division of Internal Medicine, Asthma and Allergy, Barlicki University Hospital, Medical University of Lodz, Lodz, Poland; 37 Department of Prevention of Environmental Hazards and Allergology, Medical University of Warsaw, Warsaw, Poland; 38 Center for Rhinology and Allergology, Wiesbaden, Germany; 39 Medical Faculty, CRI-Clinical Research International-Ltd, Institute of Medical Statistics, and Computational Biology, University of Cologne, Hamburg, Germany; 40 Department of Otorhinolaryngology, Head and Neck Surgery, Section of Rhinology and Allergy, University Hospital Marburg, Philipps-Universität Marburg, Marburg, Germany; 41 Department of Internal Medicine, Medical University of Graz, Graz, Austria; 42 Department of ENT, Medical University of Graz, Graz, Austria; 43 Honorary Clinical Research Fellow, Allergy and Respiratory Research Group, The University of Edinburgh, Edinburgh, UK; 44 The Usher Institute of Population Health Sciences and Informatics, The University of Edinburgh, Edinburgh, UK; 45 Skin and Allergy Hospital, Helsinki University Hospital, and University of Helsinki, Helsinki, Finland; 46 Department of Lung Diseases and Clinical Immunology, University of Turku, Terveystalo Allergy Clinic, Turku, Finland; 47 Allergy Section, Department of Internal Medicine, Hospital Vall d’Hebron, ARADyAL Research Network, Barcelona, Spain; 48 Rhinology Unit, & Smell Clinic, ENT Department, Hospital Clínic, Clinical & Experimental Respiratory Immunoallergy, IDIBAPS, CIBERES, University of Barcelona, Barcelona, Spain; 49 Pneumology and Allergy Department CIBERES and Clinical, & Experimental Respiratory Immunoallergy, IDIBAPS, University of Barcelona, Barcelona, Spain; 50 Allergy Unit “D Kalogeromitros”, 2nd Dpt of Dermatology and Venereology, National & Kapodistrian University of Athens, “Attikon” University Hospital, Athens, Greece; 51 Division of Infection, & Respiratory Medicine, Royal Manchester Children’s Hospital Immunity, University of Manchester, Manchester, UK; 52 Allergy Department, 2nd Pediatric Clinic, Athens General Children’s Hospital “P&A Kyriakou,” University of Athens, Athens, Greece; 53 Department of Otorhinolaryngology, University of Crete School of Medicine, Heraklion, Greece; 54 Allergy Department, Athens Naval Hospital, Athens, Greece; 55 Upper Airways Research Laboratory, ENT Dept, Ghent University Hospital, Ghent, Belgium; 56 Sun Yat-sen University, International Airway Research Center, Guangzou, China; 57 Academic Medical Center, Univ of Amsterdam, Dept of Otorhinolaryngology, Univ Hospitals Leuven, Leuven, The Netherlands; 58 European Forum for Research and Education in Allergy and Airway Diseases (EUFOREA), Brussels, Belgium; 59 Odense University Hospital, Department of Dermatology and Allergy Centre, Odense Research Center for Anaphylaxis (ORCA), Odense, Denmark; 60 Thermofisher Scientific, Uppsala, Sweden; 61 Department of Clinical Science and Education, Södersjukhuset, Karolinska Institutet, Sach´s Children and Youth Hospital, Södersjukhuset, Sweden; 62 Sachs’ Children and Youth Hospital, Stockholm and Institute of Environmental Medicine, Karolinska Institutet, Stockholm, Sweden; 63 Centre for Clinical Research Sörmland, Uppsala University, Eskilstuna, Sweden; 64 Peercode BV, Geldermalsen, The Netherlands; 65 Transylvania University Brasov, Brasov, Romania; 66 Department of Allergy and Immunology, Hospital Quirónsalud Bizkaia, Erandio, Spain; 67 Woolcock Institute of Medical Research, University of Sydney, Woolcock Emphysema Centre, Sydney Local Health District, Glebe, NSW Australia; 68 Nucleo de Excelencia em Asma, Federal University of Bahia, Salvador, Brazil; 69 WHO GARD Planning Group, Salvador, Brazil; 70 Division of Allergy/Immunology, University of South Florida, Tampa, FLA USA; 71 Clinica Santa Isabel, Servicio de Alergia e Immunologia, Buenos Aires, Argentina; 72 Center of Excellence in Asthma and Allergy, Médica Sur Clinical Foundation and Hospital, México City, Mexico; 73 Finnish Meteorological Institute (FMI), Helsinki, Finland; 74 Nova Southeastern University, Fort Lauderdale, FL USA; 75 Department of Medicine, Clinical Immunology and Allergy, McMaster University, Hamilton, ON Canada; 76 Department of Pulmonary Diseases, Faculty of Medicine, Celal Bayar University, Manisa, Turkey; 77 University Hospital, Montpellier, France; 78 INSERM U 1168, VIMA : Ageing and Chronic Diseases Epidemiological and Public Health Approaches, Villejuif, France; 79 Université Versailles St-Quentin-en-Yvelines, UMR-S 1168, Montigny Le Bretonneux, France; 80 Charité, Universitätsmedizin Berlin, Humboldt-Universität zu Berlin, Berlin, Germany; 81 Department of Dermatology and Allergy, Berlin Institute of Health, Comprehensive Allergy Center, Berlin, Germany; 82 Medscript, Paraparaumu, New Zealand; 83 MEDIDA, Lda, Porto, Portugal; 84 UCIBIO, REQUINTE, Faculty of Pharmacy and Competence Center on Active and Healthy Ageing of University of Porto (Porto4Ageing), Porto, Portugal; 85 Division of ENT Diseases, CLINTEC, Karolinska Institutet, Stockholm, Sweden; 86 Department of ENT Diseases, Karolinska University Hospital, Stockholm, Sweden

**Keywords:** Allergic rhinitis, Anti-histamines, Corticosteroids, ICT, Mobile health, MASK, Treatment

## Abstract

**Background:**

The analysis of mobile health (mHealth) data has generated innovative insights into improving allergic rhinitis control, but additive information is needed. A cross-sectional real-world observational study was undertaken in 17 European countries during and outside the estimated pollen season. The aim was to collect novel information including the phenotypic characteristics of the users.

**Methods:**

The *Allergy Diary*–*MASK*-*air*–mobile phone app, freely available via Google Play and App, was used to collect the data of daily visual analogue scales (VASs) for overall allergic symptoms and medication use. Fluticasone Furoate (FF), Mometasone Furoate (MF), Azelastine Fluticasone Proprionate combination (MPAzeFlu) and eight oral H1-antihistamines were studied. Phenotypic characteristics were recorded at entry. The ARIA severity score was derived from entry data. This was an a priori planned analysis.

**Results:**

9037 users filled in 70,286 days of VAS in 2016, 2017 and 2018. The ARIA severity score was lower outside than during the pollen season. Severity was similar for all treatment groups during the pollen season, and lower in the MPAzeFlu group outside the pollen season. Days with MPAzeFlu had lower VAS levels and a higher frequency of monotherapy than the other treatments during the season. Outside the season, days with MPAzeFlu also had a higher frequency of monotherapy. The number of reported days was significantly higher with MPAzeFlu during and outside the season than with MF, FF or oral H1-antihistamines.

**Conclusions:**

This study shows that the overall efficacy of treatments is similar during and outside the pollen season and indicates that medications are similarly effective during the year.

## Background

Observationl real-life studies using mobile technology can complement randomized control trials (RCTs) and improve the positioning of allergic rhinitis (AR) medications in care pathways. MASK-air (Mobile Airways Sentinel NetworK) is an information and communication technology (ICT) system which is centred around the patient. It uses a treatment scroll list which includes all medications customized for each country as well as a visual analogue scale (VAS) to assess rhinitis control [1–3]. Two studies in over 9000 users and 22 countries enabled differentiation between AR treatments [3, 4] and showed that the assessment of daily data was useful in the understanding of treatment patterns. Most allergic rhinitis (AR) patients use on-demand treatment when they are suboptimally controlled. As in resistant hypertension, defined by the number of medications used to control the disease [5], many patients have a worse control when increasing their medications [3, 4]. Differences in efficacy between intranasal corticosteroids (INCS) and intra-nasal MPAzeFlu were observed [3, 4]. These studies were carried out across the year and it is possible that the results differ during and outside the pollen season as the allergen exposure differs and the disease may not be the same in terms of phenotypes [6, 7] and costs [8]. Another MASK study in 12,143 users and 23 countries found that very few patients reporting data for several days were adherent [9]. These studies combined propose novel concepts for AR treatment. However, they failed to show certain key facts including the phenotypic characteristics of the patients at entry and whether the conclusions raised are applicable during and outside the pollen season.

The present analysis is a follow-up of previous MASK studies attempting to answer unresolved questions to provide novel real-world data information. A new cross-sectional observational study undertaken in 9037 users and 17 European countries examined AR treatments during and outside the pollen seasons (2016, 2017 and 2018). Two-thirds of the participants were already enrolled in previous studies, but analyses differed. The aim of the study was (i) to assess the participants' characteristics to better assess their phenotypes, (ii) to study whether the same trends in treatment efficacy are found during high and low allergen loads, assessed according to a recent study [10], and (iii) to investigate whether the trends in treatment efficacy were associated with the severity of the disease at entry. The study was focussed on the most commonly used intra-nasal medications containing corticosteroids: Fluticasone Furoate (FF), Mometasone Furoate (MF) and MPAzeFlu [3, 4], reported as monotherapy or co-medication [3, 4, 11]. It also focussed on the most common oral H_1_-antihistamines (OAH) reported as monotherapy: Bilastine, Cetirizine (CET), Desloratadine (DL), Ebastine, Fexofenadine (FEXO), Levocetirizine (LEVOCET), Loratadine (Lora) and Rupatadine. We did not study OAH reported as co-medication, as they are usually associated with INCS. Untreated days were used as a control group.

## Methods

### Users

All users of the app in Europe in 2016, 2017 and 2018 were included with no exclusion criteria and according to methods previously described [4, 11] .

### Setting

Users from 17 countries filled in the *Allergy Diary* (Tables  1 and 2).

**Table 1:** Country and number of users recording Visual Analogue Scale score using MASK-air^®^ during the pollen season

Country	VAS measurements (days)				
	1	2 to 7	8 to 14	> 14	Total
Austria	144 (57%)	74	14	22	254
Belgium	50 (57%)	26	6	6	88
Czech Republic	9 (29%)	10	2	10	31
Denmark	20 (38%)	18	5	10	53
Finland	109 (43%)	90	20	32	251
France	378 (56%)	222	28	43	671
Germany	205 (38%)	141	54	141	541
Greece	22 (17%)	33	21	53	129
Italy	408 (45%)	294	67	132	901
Lithuania	64 (23%)	82	37	98	281
Netherlands	341 (46%)	276	58	67	742
Poland	251 (45%)	189	35	84	559
Portugal	549 (49%)	439	60	82	1130
Spain	102 (32%)	98	39	78	317
Sweden	16 (40%)	13	6	5	40
Switzerland	428 (61%)	200	27	42	697
UK	101 (40%)	95	39	19	254
Total	3197 (46%)	2300 (33%)	518 (8%)	924 (13%)	6939

**Table 2:** Country and number of users recording Visual Analogue Scale score using MASK-air^®^ outside the pollen season

Country	VAS measurements (days)				
	1	2 to 7	8 to 14	> 14	Total
Austria	33 (54%)	15	3	10	61
Belgium	24 (46%)	17	4	7	52
Czech Republic	6 (60%)	0	0	4	10
Denmark	18 (55%)	13	0	2	33
Finland	26 (56%)	18	1	1	46
France	45 (48%)	34	4	10	93
Germany	90 (60%)	37	8	15	150
Greece	38 (31%)	35	15	35	123
Italy	139 (36%)	101	32	109	381
Lithuania	35 (20%)	51	22	67	175
Netherlands	64 (61%)	25	9	7	105
Poland	105 (58%)	53	10	14	182
Portugal	114 (50%)	76	20	19	229
Spain	95 (39%)	79	28	44	246
Sweden	26 (51%)	20	3	2	51
Switzerland	20 (71%)	29	0	0	28
UK	71 (53%)	42	9	11	133
Total	949 (45%)	624 (30%)	168 (8%)	357 (17%)	2098

### Ethics and privacy

The Allergy Diary is CE1 registered. By using k-anonymity, the data were all anonymized including the data related to geolocalization [12]. MASK-air^®^ is in line with the General Data Protection Regulation (GDPR) EU Directive 95/46/EC [13]. Independent Review Board approval was not required since the study is observational and users agree to having their data analysed (terms of use).

### Allergy diary (MASK-air^®^)

Geolocalized users self-assessed their daily symptom control using the touchscreen functionality on their smart phone to click on a VAS score (ranging from 0 to 100) for overall symptoms (global VAS). Some users reported VAS scores more than once a day. In previous studies, we found that the highest reported value should be used and we followed this. According to previous studies, severity was defined as “no symptoms” (VAS ranging from 0 to 20), “mild” (20 to 50) and “severe” (≥ 50) [1, 14].

Users input their daily medications using a scroll list which contains all country-specific OTC and prescribed medications available for each country. Both the active ingredient and the marketed OTC and prescribed medications are listed. The list has been populated using IMS data. Days with or without treatment were reported by users. The present study is another MASK study. Some of the raw data used in the other papers (up to December 2017) were used in this study [4]. However, new data have been included, many of the analyses are different and estimated allergen exposure was not previously analyzed.

### Time of the study

We did not study all individual locations as only around 60% of subjects agreed to be geolocalized and we knew only the country of origin in the non-geolocalized users. We therefore estimated the pollen season for each country using Google Trends and terms previously defined [15, 16]. We found that overall, across Europe, the season covered March 15 to the end of June. We have published a series of papers on Google Trends to better understand pollen seasons and the drawbacks of the method [15, 17–23]. They can roughly appreciate the season. On the other hand, pollen counts cannot be used on a daily basis. Moreover, they are not available for all locations. Thus, they cannot be used in the current study. To assess the pollen season precisely, personal samplers should be used but it would be impossible to use them in thousands of patients and, due to privacy, they cannot be used in this study.

We estimated the period outside the pollen season as August 1 to December 31. We therefore avoided the early tree pollen season (January-March) and excluded days recorded in Austria and France between August 1 and September 15 to avoid the ragweed pollen season. In a recent paper, the same approach was used to assess impact of pollution on the pollen season [24].

### Selection of medications

The International Nonproprietary Names (INN) classification was used for drug nomenclature [25]. Monotherapy was defined as days when only one single medication for rhinitis was reported. MPAzeFlu contains two drugs but, being a fixed combination, it was considered as monotherapy. Co-medication was defined as days with two or more medications for rhinitis. Asthma medications were not considered in co-medication.

### Characteristics at entry

According to a previous study, we considered AR symptoms recorded upon the first use of the app (rhinorrhea, sneezing, nasal congestion, nasal itching, ocular symptoms) [26]. On the same day (i.e. at entry), we assessed the ARIA severity score calculated by using the four questions regarding impact on sleep, daily activities, work/school attendance, and bothersome symptoms. Each of these four items was ascribed a score of 1 (“Yes”) or 0 (“No”). The total ARIA score ranged from 0 (no impairment) to 4 (severe impairment). This score was found to correlate with EQ-5D and WPAI-AS using MASK [27] and was used in an epidemiological study [28].

### Size of the study

In this study, all registered users were included to obtain the best possible estimates for the specified time window. From previous studies, the  numbers tested largely exceed those needed to find significant differences in the full set analysis [4].

### Stratification of the users

The stratification was determined by season of enrolment (i.e. during or outside the pollen season).

### Statistical methods

A non-Gaussian distribution was found for the data. Non-parametric tests and medians (and percentiles) were used.

### Analysis of the data

All analyses were conducted separately for users who were enrolled and used the app (i) during the pollen season (discarding days reported outside the pollen season by those users) and (ii) outside the pollen season (discarding days reported during the pollen season by those users).

All analyses were conducted by comparing the days when app users reported the use of INCS treatment (FF, MPAzeFlu, MF), the use of OAH in monotherapy, and the days when users did not report any treatment (days with other treatment were excluded from the analyses).

According to the treatment (FF, MPAzeFlu, MF, OAH in monotherapy or no treatment) reported at entry day (thereafter called Day 1), we compared (i) characteristics reported by the user on Day 1 (i.e. AR symptoms, impact of symptoms and ARIA score), (ii) the distribution of global VAS reported by the users on Day 1, and (iii) the proportion of monotherapy versus comedication reported for the use of that treatment.

The comparison analyses described in (ii) and (iii) were also conducted on all the days of App use, i.e. for all the days of App use, we compared the distribution of global VAS, as well as the proportion of monotherapy versus comedication, according to the treatment reported on that day.

Finally, for each treatment, we compared the average number of days of treatment reported per user, estimated by dividing the total number of days for which the use of a medication was reported by the total number of users reporting that medication at least once.

To investigate the consistency of our results during and outside the pollen season, we compared characteristics at entry between during and outside the pollen season.

The ARIA score on Day 1 (ranging from 0 to 4) was considered either as a continuous or a categorical variable. Global VAS was considered either as a continuous, or a categorical variable - using three cutoffs: VAS < 20/100 (controlled days), VAS 20-49 (days with moderate control), VAS ≥ 50 (days with poor control) [4, 11]. Chi square tests were used to compare the distribution of categorical variables (i.e. symptoms and impact of symptoms on Day 1, ARIA score on Day 1, global VAS categories). Kruskal–Wallis tests were used to compare the distribution of continuous variables (i.e. ARIA score, global VAS).

## Results

### Demographic characteristics

The study included 9037 users (i.e. 6939 who started to use the app during the pollen season and 2098 who started outside the season). Roughly 5% of users did not report their age or reported an age of below 10. Users ranged from zero to 91 years-old (mean, SD: 33.5 ± 15.5 years). There were 53.5% of women and 46.5% of men.

**Fig. 1:**
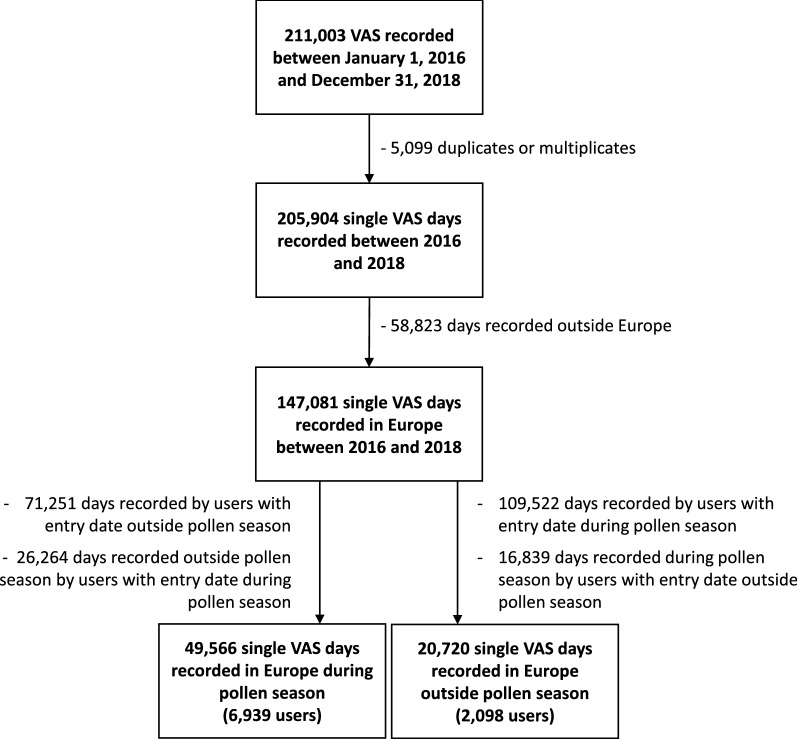
Flow chart of the study population

A total of 211,003 days were recorded between 2016 and 2018. Duplicates or multiplicates for the same day were found in 4397 days. 49,566 days were recorded by the 6939 users during the pollen season. There were 23,377 (54.4%) days without treatment and 19,568 (45.6%) days with the targetted INCS or OAH. 20,720 days were recorded by the 2098 users outside the pollen season. There were 13,130 (69.5%) days without treatment and 5756 (30.5%) days with treatment (Fig. 1).

### Characteristics on Day 1

Characteristics on Day 1 are given in Tables 3 and 4 for the pollen season and in Tables 3 and 5 for outside the pollen season.

**Table 3:** Comparison of characteristics and VAS levels at entry recorded during and outside the pollen season

		During pollen season (n = 6939)	Outside pollen season (n = 2098)	*P* value
Symptoms Day 1				
	Itchy nose (%)	73	66	<0.001
	Sneezing (%)	61	55	<0.001
	Congestion (%)	69	65	0.001
	Red eyes (%)	46	37	<0.001
	Itchy eyes (%)	68	53	<0.001
	Watery eyes (%)	47	38	<0.001
Impact of symptoms Day 1				
	Sleep (%)	38	35	0.06
	Daily activities (%)	45	39	<0.001
	Work/school (%)	30	26	<0.001
	Bothersome (%)	76	68	<0.001
ARIA score (%)	0	14	20	
	1	30	32	
	2	25	21	<0.001
	3	18	16	
	4	14	12	
	Median [p25-p75]	2 [1–3]	1 [1–3]	<0.001

*FF* Fluticasone Furoate, *FP* Fluticasone Propionate, *MF* Mometasone Furoate, *MPAzeFlu* Azelastine-Fluticasone Propionate

*Chi square tests were used for categorical variables (i.e. baseline symptoms and impact of symptoms, ARIA score); Kruskal–Wallis tests were used for continuous variables (i.e. ARIA score)

p25: 25^th^ percentile; p75: 75^th^ percentile

**Table 4:** Results for all participants recruited during the pollen season

	Treatment days				No treatment days	P value*
	FF	MPAzeFlu	MF	OAH mono		
N users Day 1	331 (5.5%)	159 (2.7%)	351 (5.9%)	1414 (23.6%)	3736 (62.4%)	
Symptoms Day 1						
Runny nose (%)	69	74	75	78	69	0.77
Itchy nose (%)	68	55	65	67	57	0.006
Sneezing (%)	79	73	76	87	77	0.20
Nasal congestion (%)	75	74	79	71	64	0.37
Red eyes (%)	50	44	42	52	42	0.56
Itchy eyes (%)	70	64	68	74	64	0.22
Watery eyes (%)	48	42	45	54	43	0.29
Impact of symptoms Day 1						
Sleep (%)	44	44	47	41	32	0.70
Daily activities (%)	41	50	50	49	41	0.36
Work/school (%)	31	42	33	33	27	0.02
Bothersome (%)	76	77	77	78	74	0.94
ARIA score (%)						
0	11	11	12	11	16	
1	29	26	23	28	32	
2	30	24	27	26	24	0.08
3	19	16	23	21	16	
4	12	23	16	15	12	
Median [p25-75]	2 [1–3]	2 [1–3]	2 [1–3]	2 [1–3]	2 [1–3]	0.36
VAS global Day 1 (%)						
N	331	159	351	1414	3736	
<20	17	18	16	16	30	0.53
20-49	32	28	33	26	28	
≥50	51	55	51	58	42	
Median [p25-75]	50 [28–71]	52 [25–73]	50 [28–68]	55 [30–75]	40 [15–66]	0.72
VAS global – all days (%)						
N days	3186	2594	4093	9780	23,377	
N users	507	256	548	2196	4569	
Average number of days per user^±^	6.3	10.1	7.5	4.5	5.1	
<20	42	55	52	48	58	0.0001
20-49	32	27	30	28	24	
≥50	26	18	18	24	18	
Median [p25-75]	26 [8–50]	16 [6–38]	19 [7–39]	21 [7–48]	14 [3–38]	0.0001

*FF* Fluticasone Furoate, *MF* Mometasone Furoate, *MPAzeFlu* Azelastine-Fluticasone Propionate

* Comparing MPAzeFlu versus FF or MF. Chi square tests were used for categorical variables (i.e. symptoms and impact of symptoms, ARIA score, global VAS categories); Kruskal–Wallis tests were used for continuous variables (i.e. ARIA score, global VAS)

^±^Estimated by dividing the total number of days for which the use of a medication was reported by the total number of users reporting that medication

p25: 25^th^ percentile; p75: 75th percentile

**Table 5:** Results for all participants recruited outside the pollen season

N users Day 1	Treatment days				No treatment days	P value*
	FF	MPAzeFlu	MF	OAH mono		
	99	80	95	275	1299	
Symptoms day 1						
Runny nose (%)	72	55	65	71	65	0.03
Itchy nose (%)	72	53	61	61	53	0.03
Sneezing (%)	76	49	71	74	67	<0.001
Nasal congestion (%)	84	63	78	65	62	0.001
Red eyes (%)	39	21	38	41	36	0.006
Itchy eyes (%)	55	38	47	62	52	0.04
Watery eyes (%)	41	29	27	45	34	0.35
Impact of symptoms day 1						
Sleep (%)	47	36	43	35	32	0.17
Daily activities (%)	46	36	34	37	38	0.54
Work/school (%)	30	31	24	25	24	0.51
Bothersome (%)	84	55	68	67	67	<0.001
ARIA score (%)						
0	8	22	19	20	23	
1	30	38	29	34	31	
2	23	16	22	21	21	0.06
3	22	10	22	12	15	
4	16	14	7	13	11	
Median [p25-75]	2 [1–3]	1 [1–2]	2 [1–3]	1 [1–3]	1 [0–2]	0.04
VAS global day 1 (%)						
N	99	80	95	275	1299	
<20	25	29	27	27	41	0.68
20-49	28	34	33	31	29	
≥50	46	38	40	42	30	
Median [p25-75]	44 [19–67]	34.5 [15–62.5]	46 [17–64]	38 [18–66]	29 [6–54]	0.25
VAS global – all days (%)						
N days	1116	1258	1437	1956	13,120	
N users	167	128	154	437	1553	
Average number of days per user^±^	6.7	9.8	9.3	4.5	8.5	
<20	50	54	59	50	74	0.0001
20-49	29	33	27	27	16	
≥50	21	13	15	23	10	
Median [p25-75]	19 [5.5-44]	18 [7–36]	14 [5–34]	19 [5-47]	5 [0-20]	0.18

*FF* Fluticasone Furoate, *MF* Mometasone Furoate, *MPAzeFlu* Azelastine-Fluticasone Propionate

* Comparing MPAzeFlu versus FF or MF. Chi square tests were used for categorical variables (i.e. symptoms and impact of symptoms, ARIA score, global VAS categories); Kruskal–Wallis tests were used for continuous variables (i.e. ARIA score, global VAS)

^±^ Estimated by dividing the total number of days for which the use of a medication was reported by the total number of users reporting that medication

p25: 25th percentile; p75: 75th percentile

During the pollen season (Table 4), 69 to 78% of users reported rhinorrhoea on Day 1. Other nasal symptoms were reported in 55 to 87% of users, and ocular symptoms in 42 to 74%. Most users reported bothersome symptoms (74–78%). Impact on sleep, daily activities and work/school attendance was reported in 27–50% of users. The ARIA score was similar in all five groups of users.

Outside the pollen season (Table 5), 55 to 72% of users reported rhinorrhoea on Day 1. Other nasal symptoms were reported in 49 to 84% of users and ocular symptoms in 21 to 62%. Most users reported bothersome symptoms (55–84%). Impact on sleep, daily activities and work/school attendance was reported in 24 to 47% of users. The use of MPAzeFlu on Day 1 was significantly associated with fewer symptoms, compared to the use of FF or MF. App users who reported the use of MPAzeFlu on Day 1 were less likely to report a severe impact of symptoms, compared to users of FF or MF on Day 1. However, the difference was only borderline significant.

Significantly more symptoms on Day 1 were reported during the pollen season than outside the pollen season, and the ARIA severity score was significantly higher outside the pollen season. Similar trends were found when restricting the population to users not reporting treatment on Day 1 (results not shown).

### Treatment efficacy

During the pollen season, on Day 1, VAS levels were reported by 3736 users without treatment, 1414 users with OAH in monotherapy and 841 users with INCS treatment (Table 4). No statistically significant difference in VAS levels was observed between INCS treatments on Day 1. When all VAS days were studied, we observed significantly lower VAS levels in MPAzeFlu days compared to other INCS (FF or MF) days (p = 0.0001, Table 4).

Outside the pollen season, on Day 1, VAS levels were reported by 1299 users without treatment, 275 users with OAH in monotherapy and 274 users with INCS treatment (Table 5). No statistically significant difference in VAS levels was observed between INCS treatments on Day 1. When all VAS days were studied, we observed non-significant differences between MPAzeFlu use compared to other INCS use or OAH.

### Monotherapy versus co-medication according to INCS use

**Fig. 2:**
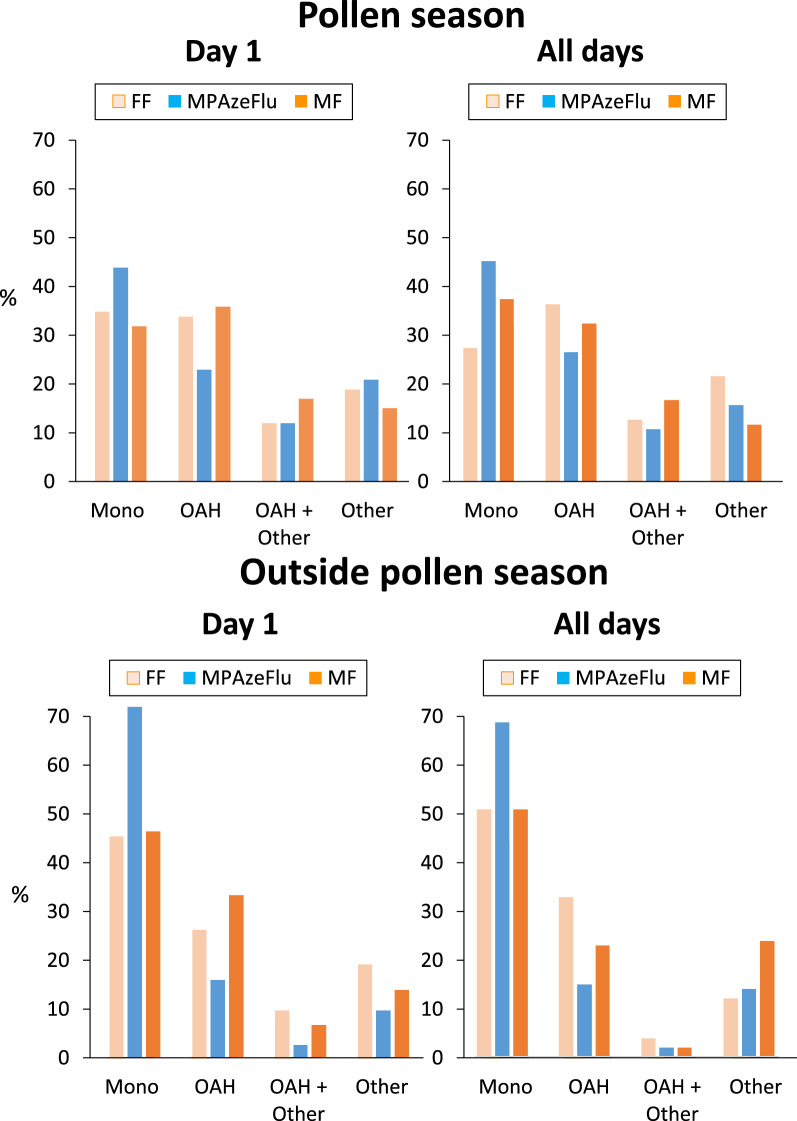
Proportion of INCS treatment groups on all days during and outside the pollen season

During the pollen season, monotherapy was significantly more reported in users who reported the use of MPAzeFlu on Day 1 (44%) compared to app users who reported the use of FF or MF on Day 1 (i.e. between 30 and 35%) (p < 0.01). Similar results were found when all VAS days were studied (p < 0.001) (Fig. 2).

Outside the pollen season, monotherapy was significantly more reported in users who reported the use of MPAzeFlu on Day 1 (71%) compared to those who reported the use of FF or MF on Day 1 (40 to 50%) (p < 0.001). Similar results were found when all VAS days were studied (p < 0.001) (Fig. 2).

### Number of days with treatment

In untreated users, the estimated average number of days of reporting per user increased from 5.1 (during pollen season) to 8.5 (outside). Both during and outside the pollen season, there was a similar trend of days reported from OAH, FF, MF to MPAzeFlu (Tables 4 and 5). The average number of days of reporting per user was low for OAH (4.5 days) and increased to around 10 days for MPAzeFlu (during and outside pollen season).

## Discussion

Two MASK studies [4, 11] have shown that, in real life, the assessment of days can provide (i) information on patients’ treatment (ii) novel insight into the behaviour of AR patients towards treatment and (iii) novel concepts for change management of AR [29]. In the present study (Table 3), we show that the ARIA severity score is (i) lower outside than during the pollen season (ii) similar for all INCS treatment groups on Day 1 during the pollen season and (iii) lower in the MPAzeFlu group than in the MF and FF groups outside the pollen season. We also show that MPAzeFlu might be more effective than FF or MF in the pollen season (lower VAS levels are reported in days with MPAzeFlu treatment, and MPAzeFlu is more frequently used as monotherapy) as well as outside the pollen season (more frequent use of monotherapy). Finally, the number of days of reported treatment per user increased from OAH to MPAzeFlu (Table 6).

**Table 6:** New information provided by this paper

1. There was no differential assessment of MASK during and between the pollen seasons
2. There was no assessment of baseline characteristics
3. Patients included in MASK have moderate/severe AR during and outside the pollen season although they were less severe outside the pollen season

### Strengths and limitations

Strengths and limitations of MASK have previously been reported [11]. As for all studies using participatory data, potential biases include (i) the likelihood of sampling bias being present (ii) the lack of generalizability of the study that was found, as bothersome symptoms are present in around 80% of users, indicating that most users have a moderate to severe disease, and (iii) outcome misclassification that cannot be assessed. Data obtained with an app are not representative of the general population.

In the previous MASK studies, there was very little information on patient characteristics. In the present study, we examined characteristics at entry in more detail.

As in other studies [4, 11], we used days in a cross-sectional analysis because there is no clear pattern of treatment and a longitudinal study was not feasible since users mostly use the App intermittently.

In the current study, we cannot ascertain that the users are allergic to a given allergen since this information is not available for all patients. Moreover, we did not assess the real pollen exposure of the patients, as only 60% of them agreed to be geolocated.

The diagnosis of AR was not supported by a physician but was a response to the question: “Do you have allergic rhinitis? Yes/No”. Some of the users with non-allergic rhinitis may therefore have responded “Yes” to the question. However, > 95% of responders declared symptoms of AR by questionnaire. Precise patient characterization is impossible using an App, but every observational study using MASK was able to identify days with poor control or criteria of severity [26, 27, 30–32]. Moreover, some data are highly similar across studies. These include the percentage of untreated days (i.e. approximately 50% of the total days recorded).

There is a clear deviation in the results obtained in highly populated countries and a very high prevalence of allergic rhinitis with little collection data. These results could possibly influence the data.

The current study has many strengths including larger numbers, multiple countries, range of treatments studied and patient/person-generated data.

### Interpretation of the results and generalizability

This real-world assessment of the *Allergy Diary* using VAS allows the  assessment of treatment efficacy by days [4, 11]. This observational study complements the two previous studies in many aspects (Table 7).

**Table 7:** Key messages

1. What is already known about this topic? The MASK mHealth App has generated real-world evidence that has led to novel pharmacotherapy insights – for example, that patterns of treatment for allergic rhinitis do not always accord with guidelines.
2. What does this article add to our knowledge? Results can be extended to both the estimated pollen season and the period outside. The study shows that rhinitis medications are equally effective during and outside the pollen season. The baseline characteristics of the patients show that most users have moderate to severe rhinitis and that mHealth data may not be generalisable to all patients with allergic rhinitis
3. How does this study impact current management guidelines? This paper confirms the importance of the MASK mHealth App in next-generation GRADE guidelines that embed real-world-evidence into the GRADE-based evidence. The same treatment can be administered during and outside the pollen season

First, it shows that over 75% of patients using the app during the pollen season have bothersome symptoms. Outside of the pollen season, the rate of bothersome symptoms is around 65%. It is therefore likely that most App users have moderate/severe AR and do not therefore represent the general population [33]. It is interesting to note that these levels of impairment are close to those of patients consulting in primary [34] or specialist care [35]. Although the impact of AR is less important outside the pollen season than during, differences are not very important in the ARIA score.

Second, it was expected that MPAzeFlu would have been given to more severe patients. The ARIA score was not different between groups in the pollen season. In contradistinction, the ARIA score was significantly lower outside the pollen season in untreated users and even lower in the MPAzeFlu users.

Third, both during and outside the pollen season, MPAzeFlu is associated with less symptoms, something that seems consistent with being the most potent medication in a randomized controlled trial [36]. However, there are differences between seasons. During the pollen season, the use of MPAzeFlu is associated with the lowest VAS levels in treated groups, and MPAzeFlu is used more commonly as monotherapy. Outside of the pollen season, all medications appear to be associated with similar VAS levels. However, MPAzeFlu is used as a monotherapy in 70% of days whereas the other INCS are used in less than 50% of days. Nevertheless, given the cross-sectional setting of our study, effectiveness cannot be inferred easily.

Fourth, the estimated average number of days reported per user in the MPAzeFlu group was almost twice as high as that among the OAH group. Although there is no simple interpretation, it is suggested that the most effective treatments are reported for a longer period of time. However, we cannot assess duration in this cross-sectional setting, but this finding is consistently found across MASK studies [11]. Again, there is no major difference between seasons.

Fifth, as already found in all users [4, 11], median VAS levels are the lowest in untreated days, both during and outside the pollen season. This can be interpreted as subjects using treatment when they do not feel well, in opposition to the paradigm in which those who take medication are the ones with controlled symptoms (and therefore lower VAS). Also, the patterns of co-medication of MPAzeFlu by comparison to FF or MF are similar in the two periods.

Sixth, the behaviour of users appears to be quite similar between seasons. In particular, they report the same number of days with the same medications.

This study shows that, in real-life, the same treatments have similar patterns during and outside the pollen season for most criteria tested. This is an important finding that may impact guidelines considering AR severity rather than seasonal patterns [37, 38].

## Conclusions

Although the MASK mHealth App has generated real-world evidence that has led to novel pharmacotherapy insights, the current study extends our knowledge by (i) assessing the characteristics of the patients, (ii) showing that results can be extended to both the estimated pollen season and the period outside the season, and (iii) showing that rhinitis medications are equally effective during and outside the pollen season (Table 5).

Real-world data (RWD) and real-world evidence (RWE) both play an increasing role in health care decisions supporting clinical trial designs and observational studies to generate innovative and new treatment approaches. This study shows that the overall efficacy of treatments is similar during and outside the pollen season and indicates that medications are similarly effective during the year. It is an important study for the digital transformation of health and care in rhinitis and asthma multimorbidity [3, 39–41].

## Data Availability

On request to Kyomed Innov. Members of the MASK study group have free access to the data.

## Abbreviations

AR: Allergic rhinitis
ARIA: Allergic Rhinitis and its Impact on Asthma
MPAzeFlu: Intranasal azelastine-fluticasone propionate
CET: Cetirizine
DL: Desloratadine
FEXO: Fexofenadine
FF: Fluticasone Furoate
FP: Fluticasone Propionate
GRADE: Grading of Recommendations, Assessment, Development and Evaluation
ICT: Information Communication Technology
INCS: Intranasal corticosteroid
INN: International Nonproprietary Names
LEVOCET: Levocetirizine
Lora: Loratadine
MASK-rhinitis: Mobile Airways Sentinel NetworK for allergic rhinitis
MF: Mometasone Furoate
OAH: Oral H_1_-anti-histamine
RCT: Randomized controlled trial
VAS: visual analogue scale
