# Corrigendum: Treatment of allergic rhinitis during and outside the pollen season using mobile technology. A MASK study

**DOI:** 10.1002/clt2.12126

**Published:** 2022-03-03

**Authors:** Bousquet Jean

1

In [1], the following errors were published in the author by‐line and affiliations on page 12.

The author names and their correct affiliations are listed below.

E. Eller^60^, I. Kull^61^, E. Melén^61^, M. Wickman^62^, G. De Vries^63^, M. van Eerd^63^, I. Agache^64^, I. J. Ansotegui^65^, S. Bosnic‐Anticevich^66^

^**6**0^Odense Research Center for Anaphylaxis, ORCA, Dept. Dermatology and Allergy Center, Odense University hospital, Denmark.

^61^Department of Clinical Science and Education, Södersjukhuset, Karolinska Institutet, Sach's Children and Youth Hospital, Södersjukhuset, Sweden.

^62^Centre for Clinical Research Sörmland, Uppsala University, Eskilstuna, Sweden.

^63^Peercode BV, Geldermalsen, The Netherlands.

^64^Transylvania University Brasov, Brasov, Romania.

^65^Department of Allergy and Immunology, Hospital Quirónsalud Bizkaia, Erandio, Spain.

^66^Quality Use of Respiratory Medicine Group, Woolcock Institute of Medical Research, The University of Sydney, and Sydney Local Health District, Sydney, NSW, Australia.
